# Spatial and temporal relationship between native mammals and free-roaming dogs in a protected area surrounded by a metropolis

**DOI:** 10.1038/s41598-019-44474-y

**Published:** 2019-06-03

**Authors:** Shih-Ching Yen, Yu-Ten Ju, Pei-Jen Lee Shaner, Hsiang Ling Chen

**Affiliations:** 1 Center for General Education, National Tsing Hua University, No. 101, Section 2, Kuang-Fu Road, Hsinchu, 300 Taiwan; 2 Department of Animal Science and Technology, National Taiwan University, 50, Lane 155, Sec. 3, Keelung Rd., Taipei, 106 Taiwan; 3 School of Life Science, National Taiwan Normal University, 88, Sec. 4, Ting-Chow Rd, Taipei, 116 Taiwan; 4 Department of Forestry, National Chung Hsing University, 145, Xingda Rd., Taichung, 402 Taiwan

**Keywords:** Conservation biology, Invasive species

## Abstract

With rapid urbanization worldwide, anthropogenic impacts such as human settlements and invasive carnivores (dogs *Canis familiaris*, cats *Felis catus*) are altering spatial distributions and temporal activity patterns of native species. In this study, we focused on spatiotemporal responses of native mammals to anthropogenic impacts in a protected area surrounded by a large metropolis (i.e. Yangmingshan National Park inside Taipei-Keelung metropolis in northern Taiwan). We collected site use data of 11 mammal species (i.e. dogs, cats, nine native species) between 2012 and 2017 with a camera system comprising 121 camera sites. We quantified anthropogenic disturbances as distance to human settlements and activity levels of free-roaming dogs and cats. Species richness and occurrences of the native mammals increased with increasing distances to human settlements and decreasing activity level of dogs, with the latter having a stronger effect than the former. Diel activity overlap between native mammals and dogs was lower during April–July season, coinciding with the breeding season for several native mammals. In contrast, activity level of cats showed no relationships with species richness, occurrences or diel activities of the native mammals. This study demonstrated negative impacts of human settlements and free-roaming dogs on native mammal communities for protected areas in urban environments, and highlights dog activity as a major anthropogenic threat to wildlife.

## Introduction

Anthropogenic habitat destruction poses serious threats to global biodiversity and is considered as a primary cause of the present extinction crisis^1^. Creating and maintaining protected areas is one of the most important tools to mitigate the impacts of habitat loss and fragmentation on biodiversity^2,3^. However, approximately one third of protected lands globally is under intense human pressure^4^. The rapid development of human settlements and tourism within and surrounding protected areas could jeopardize their effectiveness as a conservation tool^5^.

With increasing human settlements, invasive carnivores are becoming a major concern. Human presence often introduces two invasive carnivores: dogs (*Canis familiaris*)^6^ and cats (*Felis catus*)^7^. Dogs and cats are opportunistic predators that exploit a wide variety of prey^8,9^. Free-roaming dogs and cats are distributed worldwide and have caused extinctions and declines of many native species^10,11^ through predation, competition and disease transmission^12,13^. Predation by dogs is a major threat to many endangered species, and has been suggested to be the cause of 11 vertebrate species extinctions^10^. Cats might have directly or indirectly caused the extinction of at least 33 mammalian species^14^, and are estimated to kill 2.4 billion and 272 million birds per year in the U.S.^15^ and Australia^11^, respectively. The presence of dogs and cats can have a wide range of impacts on behavior and fitness of native species, such as increased vigilance^16^, decreased food intake^17^, altered site occupancies^7^ and activity patterns^18^, and reduced reproductive success^19^. These changes in behavior and fitness may lead to lowered abundance or reduced distribution of wildlife populations^7,18^. Therefore, from conservation and management perspective, it is important to monitor whether and to what extent native species are responding to the presence of dogs and cats^20^.

**Figure 1:**
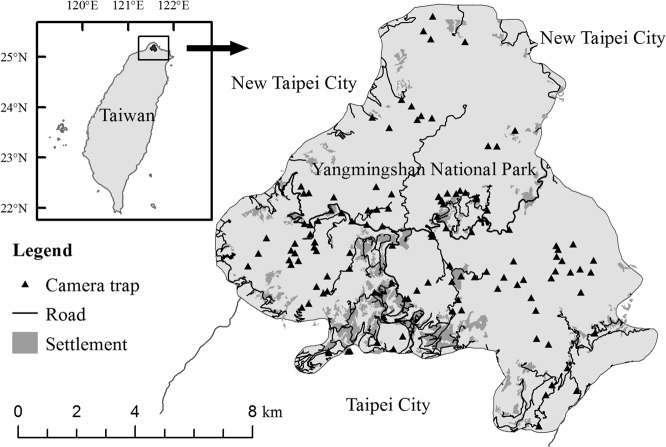
Layout of the camera system in relation to locations of human settlements in Yangmingshan National Park, Taiwan.

Taiwan is among the most densely populated regions in East and Southeast Asia, a geographic area of high conservation priority in the world given its high biodiversity coupled with rapid habitat loss from growing human population^3,21^. Despite having a comprehensive protected-area system, several protected areas in Taiwan are facing extensive anthropogenic disturbances from pre-existing human settlements, invasive carnivores, roads, and tourism. In this study, we focused on Yangmingshan National Park (YMSNP; Fig. 1), a protected area surrounded by a large metropolis where 7 million people reside. One of the park’s missions is to conserve lowland ecosystems in Taiwan, which has become increasingly challenging due to its close proximity to the metropolis. Approximately 12,000 people live inside YMSMP, and 19 million tourists visit the park annually^22^. Furthermore, the park has approximately 1,000 free-roaming dogs and at least 400 free-roaming cats (i.e. free-roaming dogs and cats are those either unowned or owned but not confined to a prescribed indoor/outdoor area)^17^. While the estimated number of dogs is close to the actual population size, the number of cats is likely underestimated because only owned cats were included^23,24^.

We collected site use data of dogs, cats and nine species of native mammals (Chinese hare *Lepus sinensis*, Chinese ferret badger *Melogale moschata*, Chinese pangolin *Manis pentadactyla*, small Indian civet *Viverricula indica*, masked palm civet *Paguma larvata*, wild boar *Sus scrofa*, Reeves’s muntjac *Muniacus reevesi*, sambar *Rusa unicolor*, Formosan macaques *Macaca cyclopis*; all medium to large-sized mammals with body mass >1 kg) between 2012 and 2017 with a remote camera system comprising 121 camera sites (Fig. 1). We used multi-species hierarchical occupancy modelling^25^ and kernel density estimation (KDE)^26^ to estimate spatial occurrences and temporal activities of the mammals, and evaluate how the native mammals respond to human settlements and activity levels of dogs and cats. We hypothesize that species richness and occurrences of the native mammals increase with increasing distance to human settlements and decreasing activity levels of dogs and cats. Furthermore, although most native mammals are nocturnal with a limited ability to shift their diel activities in response to dogs and cats, we do predict that the level of diel activity overlap between native mammals and dogs or cats to exhibit some seasonal or species-specific variations. In particular, some adjustments in diel activities may help lower temporal overlap in site use with dogs or cats, thereby reduce predation risk of the young during breeding seasons or of the adults for smaller-sized mammals.

## Results

### Effects of human settlements and invasive carnivores on occurrences and detections of the native mammals

We recorded 5,975 detections of the nine species of native mammals over 17,310 trap nights across the 121 camera sites (Supplementary Information 1). The estimated mean species richness was 3.97 (SD = 1.67; 95% confidence intervals [CI]: 3.66–4.27). The mean probability of occurrence across all species (mean community-level occurrence) was 0.40 (SD 0.13; 95% CI: 0.16–0.69), ranging from 0.06 for Chinese hare to 0.76 for Chinese ferret badgers. The mean probability of detection across all species (mean community-level detection) was 0.24 (SD = 0.06; 95% CI: 0.13–0.38), ranging from 0.06 for Chinese pangolins to 0.63 for Reeves’s muntjac (Supplementary Information 2).

**Figure 2:**
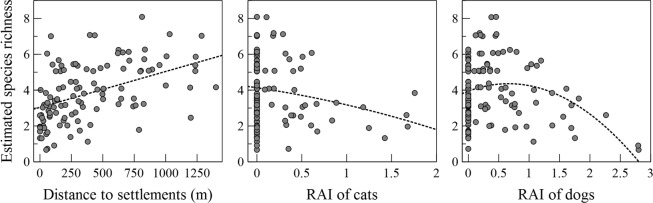
Site-specific estimates of species richness in response to anthropogenic effects. From left to right: distance to settlements (m), relative activity level index (RAI, the number of independent captures per 100 trap-nights) of dogs, and RAI of cats. The RAI in this figure were log-transformed values, log(RAI + 1).

**Figure 3:**
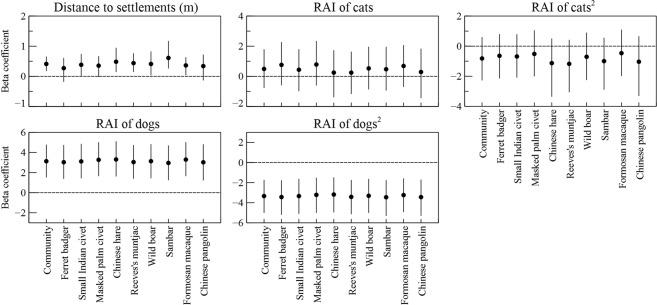
Standardized beta coefficients and their 95% confidence intervals for the anthropogenic effects: distance to settlements (m), relative activity level index (RAI, the number of independent captures per 100 trap-nights) of dogs, and RAI of cats, on the probability of occurrence by the mammal community or by a given mammal species.

Community-level occurrences, species-specific occurrences and species richness of the native mammals increased with increasing distance to human settlements and decreasing activity level (relative activity level index, RAI, quantified as the number of independent camera detections per 100 trap-nights) of free-roaming dogs. In contrast, the activity level of free-roaming cats did not affect occurrences of the native mammals at either community or species level (Table 1, Figs 2 and 3; Supplementary Information 3). For detection probability (i.e. intensity of use by animals), Reconyx cameras had lower detection probabilities at community level than Keep guard cameras (Table 1). However, the activity levels of dogs and cats did not affect detection probabilities of the native mammals (Table 1, Supplementary Information 4).

**Table 1:** Estimated means and their 95% confidence intervals for community-level hyper-parameters hypothesized to influence the probabilities of occurrence and detection of nine native mammals in Yangmingshan National Park, Taiwan 2012–2017.

Variables	Mean	SD	95% CI	
**Occurrence**				
Distance to human settlement (m)	0.41	0.13	0.17	0.65
RAI of dogs	3.13	0.85	1.51	4.78
RAI of dogs^2^	−3.34	0.87	−5.01	−1.72
RAI of cats	0.49	0.66	−0.77	1.80
RAI of cats^2^	−0.82	0.74	−2.28	0.60
**Detection**				
Cuddeback	−0.25	0.24	−0.73	0.26
Reconyx	−0.37	0.16	−0.67	−0.03
RAI of dogs	0.11	0.20	−0.27	0.53
RAI of cats	0.43	0.43	−0.38	1.37

Camera brands Cuddeback and Reconyx are compared to Keep Guard.

### Diel activity overlap between invasive carnivores and native mammals

**Figure 4:**
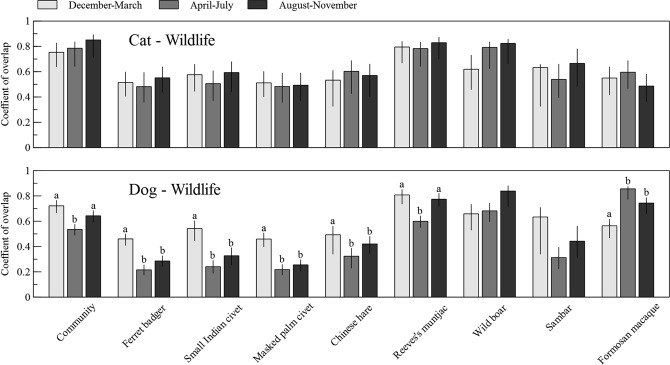
Seasonal patterns in diel activity overlap between an invasive carnivore (dogs or cats) and a native mammal. Different letters indicate a significant difference in the level of overlap between seasons. The error bars denote the 95% confidence intervals of the mean overlap.

The dogs were mainly active during daytime and the cats were active all day, while they both had activity peaks during dawn and dusk (Supplementary Information 5). Both dogs and cats did not exhibit strong seasonal variation in activity level (dogs: RAI_April–July_ = 1.48, RAI_August–November_ = 1.25, RAI_December–March_ = 0.91; cats: RAI_April–July_ = 0.64, RAI_August–November_ = 0.43, RAI_December–March_ = 0.37; P > 0.05, Mann-Whitney U tests for all pairs of seasons for dogs and cats separately). However, the native mammal community as a whole, as well as six of the eight species examined, showed seasonal differences in level of diel activity overlap (Δ) with the dogs, but not with the cats (Fig. 4). The native mammal community exhibited a lower diel activity overlap with dogs during April–July than the other two seasons, which coincides with the breeding seasons for some of the mammal species. Four of the six species that showed seasonal variation in diel activity overlaps with dogs are relatively smaller-sized (Chinese hare, Chinese ferret badgers, small Indian civets, masked palm civets), and exhibited a consistent pattern of lower diel activity overlaps with dogs during April–July and August–November than during December–March (Fig. 4). Furthermore, at community level, the seasonal Δ between dogs and native mammals ranged between 0.53 and 0.72, generally lower than that between cats and native mammals (0.75–0.85) (Fig. 4). At species level, the seasonal Δ between dogs and individual mammal species (0.22–0.86) were more varied than those between cats and individual species (0.48–0.83) (Fig. 4).

## Discussion

This study demonstrated negative anthropogenic impacts on native mammals in a protected area surrounded by a metropolis. Our findings suggest that free-roaming dogs outweigh human settlements in reducing species richness and occurrences of native mammals. In addition, the native mammals could be more sensitive to the presence of dogs than that of cats, with more reduced spatial occurrences and more varied temporal activity patterns in the presence of dogs. In central and south America, a dog population density as low as 1–4 dogs/km^2^ was sufficient to alter the distributions and activity patterns of native wildlife^18,27^. In YMSNP, dog population density could reach 30 dogs/km^2^, and the dogs were reported to roam as far as 10 km away from villages^24,28^, suggesting the negative impacts of dogs on native wildlife could be substantial and widespread.

Multi-species hierarchical occupancy models are particularly useful for assessing biodiversity and modeling occupancy of rare species, which sufficient data for single species occupancy modeling is often lacking^29,30^, such as Chinese pangolin, sambar, and Chinese hare in this study. However, we also recognize that the results for individual species derived from community level occupancy models should be interpreted with caution, because individual species are treated as random effects derived from a normally distributed, community-level hyper-parameter. Therefore, we may not be able to capture the variation of responses to anthropogenic disturbance among species, and result in the uniformity of the modeled effects of dogs across the native mammals. We acknowledge that there is a potential of mis-interpretating species-level responses, especially for species with small number of detections. For example, effects of dogs on species with large body size such as sambar deer may not as strong as for small Indian civets. Nevertheless, we believe the overall community responses should be robust. Previous studies have reported dog attacks on both ground and tree dwelling native species with a range of body size in YMSNP, including small Indian civets, wild boar, red-bellied tree squirrels (*Callosciurus erythraeus*), and Taiwan bamboo partridge (*Bambusicola sonorivox*)^31,32^. Activity of dogs might be perceived and used by the different species of native mammals as a generic risk cue^33^, and thus respond similarly.

Wildlife have different responses to human settlements. Some species may avoid human settlements due to noise, light, and presence of people^23,34^. Other species, especially those that are smaller in body size, may prefer human settlements as predator-free space (native predators are usually eradicated from human settlements^35^). The dominant predators in YMSNP are likely dogs and cats, whose abundances and activities are often associated with human settlements. Despite that we did not find correlations between distance to human settlements and RAI of invasive carnivores in this study, their effects on wildlife could still be synergistic^20^. To disentangle the effects of human settlements from that of invasive carnivores, a more complex experimental design than the current study is needed. For example, where there are dog control programs, one can compare the responses of native mammals between dog-removal versus no-removal areas with various distances to human settlements.

Although each species examined in this study could have seasonal diel activities based on their own life history and ecology, several lines of evidence suggest that dog presence could play a role. First, the native mammals showed more varied diel activity overlaps across seasons with dogs, but not cats. Second, the native mammal community exhibited a significantly lower level of diel activity overlap with dogs during the April–July season, which coincides with the breeding season for at least four native mammal species in YMSNP (Chinese ferret badgers^36^, small Indian civets^37^, masked palm civets^38^, sambar^39^). It is plausible that the native mammals employed temporal avoidance to reduce the probability of dog preying on their young. Finally, at the species level, only the two largest species (sambar and wild boar) did not showed seasonal changes in their diel activity overlaps with dogs, suggesting dog predation on smaller-sized species might influence their diel activity patterns. Nevertheless, whether the native mammals are adjusting diel activities in response to dog presence, and to what extent such strategies affect their survival and reproduction, will require more detailed behavioral and life-history data.

Invasive carnivores are known to replace native apex predators, such as dingo replacing thylacine (*Thylacinus cynocephalus*) in Australia^12^. However, invasive carnivores are unlikely to play the same functional role as native apex predators. For instance, dogs form packs and can reach a very high density due to food subsidies from human^17^. Pack hunting may lead to high predation success, and subsidized food resources may break down predator-prey regulation, increasing the chance for the predators to exceed their carrying capacity and render the ecosystem unstable^17,27^. On the other hand, cats can be inefficient predators of larger-sized prey compared to native apex predators. Interspecific killing among mammalian species occurs more frequently for pairs of species with large body-size differences^40^. Of the nine mammal species examined, only Chinese ferret badgers and Chinese hare are smaller in size than cats. Therefore, neither dogs nor cats are likely to play the functional role of native apex predators that are currently missing from Taiwan’s ecosystems.

Cats prey on birds, invertebrates and small mammals such as rodents and shrews^41^. Therefore, even if they do not act as predators of native mammals, they could be strong competitors for these shared prey. Furthermore, both cats and dogs can influence native mammals through disease transmission^42,43^. Therefore, studies on a broader spectrum of prey taxa and different types of interspecific interactions (e.g. predation, competition, disease transmission) between native wildlife and invasive carnivores are urgently needed.

Our results have immediate management implications for protected areas in urban environments, especially those with established dog populations. Specifically, we showed that species richness of native mammals seemed to drop after dog activities exceeded some threshold level, and diel activity overlaps between native mammals and dogs varied across seasons and species. Therefore, we recommend the management to focus on identifying and controlling for a target dog number, particularly during the breeding seasons of native mammals and/or in areas where smaller-sized mammals are most abundant. To manage a target number of free-roaming dogs in a protected area, removal is the most ideal solution^13,44^. However, for protected areas near urban areas, such as YMSNP, the effectiveness of dog removal program might be limited. Dog abandonment occurs occasionally, and euthanasia is extremely unpopular among the public given people’s emotional ties to dogs^45^. Therefore, to reach the target dog number, the management should also consider tools such as public education in pet owner responsibility (e.g. no abandonment, routine sterilizing and proper confinement) and transferring captured dogs to existing shelters to minimize the need for euthanasia.

Global domestic dog population abundance is over 700 million^13^, making the protected areas in close proximity to urban environments vulnerable to the risk of free-roaming dogs. The YMNSP case is by no means unique. This study provided quantitative evidence on spatial and temporal responses of native mammals to anthropogenic impacts, particularly in the form of free-roaming dogs. Furthermore, our findings can inform management practices regarding dog control in protected areas. Finally, we demonstrated that the use of a remote camera system, in combination with occupancy modeling, can provide routine, scientific data on spatial and temporal interactions between invasive carnivores and native wildlife. We believe such monitoring programs will become increasingly important to guide our efforts to mitigate anthropogenic impacts on wildlife in areas undergoing rapid urbanization.

## Methods

### Study area

The Yangmingshan National Park is located in the center of Taipei–Keelung metropolitan area of northern Taiwan (Fig. 1). The park was established in 1985, encompassing an area of 113 km^2^ with an elevation range of 56–1,120 m. We classified a year into three seasons: December–March (a colder season with a mean temperature of 13.2 °C, and mean monthly precipitation of 224 mm), April–July (a hotter and dryer season with a mean temperature of 22.4 °C, and a mean monthly precipitation of 274 mm), and August–November (a hotter and wetter season with a mean temperature of 21.3 °C, and a mean monthly precipitation of 406 mm) according to the meteorological data at Anbu weather station (2012–2016). Lowland evergreen broad-leaved forests characterized with *Machilus* sp. and *Acacia* sp. account for 76% of the park’s vegetation cover, followed by agriculture cover (7%), herbaceous cover (6%), broad-leaved thickets (4%), plantation forests (4%), and others (3%; Hsu *et al*. 2008). At least 26 species of mammals occur in the park^46^, including seven endemic species to Taiwan (27% endemism). This study included nine native species with larger size (>1 kg). Among them, three are currently protected under Wildlife Conservation Act of Taiwan (http://conservation.forest.gov.tw), including Formosan pangolin (*M. pentadactyla pentadactyla*), a subspecies of the Critically Endangered Chinese pangolins on the IUCN Red List^47^, as well as small Indian civets, and sambar. The other six species are Formosan macaques, Reeves’s muntjac, wild boar, masked palm civets, Chinese hares and Chinese ferret badgers. Native apex predators, such as leopard cats (*Prionailurus bengalensis*) and black bears (*Ursus thibetanus*) do not occur in this area^46^.

### Camera trapping

Remote cameras are ideal tools for tracking medium- to large-sized mammals and assessing anthropogenic impacts^48^. We deployed cameras (Cuddeback Capture, Cuddeback Digital, Wisconsin, USA; HC500 and PC800, Reconyx Inc. Wisconsin, USA; KeepGuard KG780NV, Keeptime Inc., Shenzhen, China) at 121 sites throughout the study area from March 2012 to July 2017 (Fig. 1). We overlaid the study area with a 1 × 1 km grid system and placed cameras in every other grid. The three brands of cameras were randomly distributed. A total of 61 grids (41% of all grids) were sampled. More than one camera sites were sampled at 33 grids, but the cameras were not deployed simultaneously. At each camera site, cameras were active for 143 days (SE = 11) on average. We installed cameras near existing animal trails to maximize detection probability. Thus, our protocol represents a balance between systematic sampling and intentional selection of animals^49^. Cameras were set 30–80 cm from the ground with an angle slightly downward, which was expected to work well for medium- to large-sized mammals. We checked each station every month to change memory cards and batteries. Camera trap data between 2012 and 2014 were used for a previous study^32^, which focused on the population status of small Indian civets and described their potential threat from dogs.

Many environmental conditions could influence species richness and occurrence of mammals. For each camera station, we recorded vegetation cover and classified it into three types: herbaceous vegetation and fargesias thickets, broad-leaved forests, and broadleaf-conifer mixed forests. We used ArcMap 10.1 (ESRI Redlands, CA, USA) to calculate distances to the nearest human settlement and water body. The layer of human settlements, including buildings, farms and recreation areas, was extracted from a land-cover layer provided by YMSNP. The water layer was provided by the Water Resources Agency in Taiwan (https://data.gov.tw, accessed 1 Feb 2018). Elevation, slope, and solar radiation were derived from a 20-m resolution Digital Elevation Model provided by the Ministry of the Interior, Taiwan (https://data.gov.tw, accessed 1 Feb 2018).

We summarized trap-nights of effort for every camera after subtracting days where cameras malfunctioned or ran out of batteries. We used a RAI to quantify activity level of the invasive carnivores^50^. Consecutive camera detections of the same species at the same site were deemed independent when there was at least a 0.5-h interval between them or when animals could be individually distinguished^51^.

### Multi-species hierarchical occupancy modeling

We used multi-species hierarchical occupancy modeling^29,30,52^ with a Bayesian approach^25^ (Supplementary Information 6) to estimate species richness as a function of model-based estimators of species-specific occurrence at each camera site. Multi-species hierarchical occupancy model combines information across species and reduces the number of parameter estimates, while allowing for species-specific responses to covariates^53,54^. Several species in the study areas can have home ranges covering several camera sites, and likely violated the assumption of population closure. Thus, we estimated occurrence of a species at a camera site instead of occupancy, as the probability of the species using the site during the sampling period^55^.

We hypothesized the occurrences of native mammals are influenced by distance to human settlements and activity levels of dogs and cats. However, because dogs and cats are mobile, their presence could also influence detection probability (frequency of uses by the animal at a given site^56–58^, a metric of intensity of site use) of native mammals in trail camera survey. Therefore, we assumed that detection probability is affected by activity levels of dogs and cats, as well as camera brands and season. We performed preliminary LOESS regressions to explore the most likely forms of relationships between species richness of the native mammals and distance to human settlements, dog RAI or cat RAI. The patterns suggest that species richness of native mammals might have a peak around 0–0.5 RAIs of dogs and cats, which persisted even after the data points with zero RAIs were removed (Supplementary Information 7). On the other hand, species richness of native mammals appeared to have a linear relationship with distance to human settlements (Supplementary Information 7). We thus chose to apply quadratic relationships between RAI of the invasive carnivores and occurrences of native mammals.

For other covariates that may influence wildlife occupancy and detection but are not of our interests, we used a stepwise selection strategy to decide whether to retain it^59^. Specifically, a covariate was retained if its 95% CI did not include zero. For model of detection probability, we first included both effects of season and camera brands (Keep Guard [reference level], Cuddeback, Reconyx) in the model. We then removed variables that do not have significant effects. Our final base model of detection probability only included effects of camera brands to account for differences in sensitivity and trigger speed. For environmental variables that may influence occurrences, such as vegetation type, elevation, slope, solar radiation and distance to water, we included these variables sequentially to the base occupancy model that only include variables for detection probability. After the selection of the environmental covariates, which resulted in no environmental covariate being selected, we added the three variables of anthropogenic disturbances (i.e. distance to the nearest human settlement, RAI of dogs, RAI of cats) to form the final models:

$$\begin{array}{rcl}{\rm{logit}}({\rm{probability}}\,{\rm{of}}\,{\rm{occurrence}}) & = & {\rm{\alpha }}0+{\rm{\alpha }}1({\rm{distance}}\,{\rm{to}}\,{\rm{human}}\,{\rm{settlement}})\\  &  & +\,{\rm{\alpha }}2({\rm{RAI}}\,{\rm{of}}\,{\rm{dogs}})+{\rm{\alpha }}3({\rm{RAI}}\,{\rm{of}}\,{{\rm{dogs}}}^{{\rm{2}}})\\  &  & +\,{\rm{\alpha }}4({\rm{RAI}}\,{\rm{of}}\,{\rm{cats}})+{\rm{\alpha }}5({\rm{RAI}}\,{\rm{of}}\,{{\rm{cats}}}^{{\rm{2}}})\end{array}$$

$$\begin{array}{rcl}{\rm{logit}}\,({\rm{detection}}\,{\rm{probability}}) & = & {\rm{\beta }}0+{\rm{\beta }}1({\rm{Cuddeback}})\\  &  & +\,{\rm{\beta }}2({\rm{Reconyx}})+{\rm{\beta }}3\,({\rm{RAI}}\,{\rm{of}}\,{\rm{dogs}})+{\rm{\beta }}4({\rm{RAI}}\,{\rm{of}}\,{\rm{cats}})\end{array}$$

The distance to human settlement, dog RAI, and cat RAI are not correlated (Pearson’s correlation, *p* > 0.05, r < |0.3|). The RAI of dogs and cats were log-transformed. We standardized all continuous covariates to have a mean of zero and standard deviation of one before running the occupancy model.

We estimated posterior distributions of parameters using Markov chain Monte Carlo (MCMC) implemented in JAGS (version 3.4.0) through R2Jags^60^ in program R (version 3.4.2, R Foundation for Statistical Computing, Vienna, Austria). For each model, we generated three chains of 50,000 iterations after a burn-in of 10,000 and thinned by 50. We assessed convergence using the Gelman–Rubin statistic where values < 1.1 indicate convergence^61^. We used probability of occurrence matrices generated by MCMC iterations to estimate overall species richness and richness at each camera station (Supplementary Information 8)^25,30^.

### Diel activity overlap

To study the temporal relationships between native mammals and invasive carnivores, we inspected how the levels of diel activity overlap changed in response to seasonal activity levels of dogs and cats. We first calculated RAI of dogs and cats for each season to examine the variation of their seasonal activity levels. We then estimated the overlap in diel activity between native mammals and invasive carnivores in each season (except for Chinese pangolins due to its small sample size with only nine detections; Supplementary Information 1).

Seasonal RAI was calculated as the average monthly RAI of the season. We used the data from only 31 out of the 121 cameras, which were active for at least two seasons and at least 60 days per season. This helps reduce the confounding effects from spatial heterogeneity when comparing seasonal patterns based on the data taken at different locations.

We considered photographic captures of a species as a random sample derived from the distribution of its underlying continuous activity, and the diel activity describes the probability of a species being detected at any particular interval of the day. To estimate the overlap in diel activity, i.e. the similarity between two activity patterns^26^, we pooled data which were collected from all 121 camera traps and across all years. We applied KDE to calculate the Δ between species pairs^26^. The value of Δ would be between zero and one. A value closer to one indicates a higher level of overlap while a value closer to zero a lower level of overlap. The previous study^26^ suggested that the estimator “Δ_1_” (overlap evaluated for the same set of sample times between species) and “Δ_4_” (overlap evaluated separately for different sets of sample times between species, and then averaged) had good performance for smaller and larger sample size, respectively. Therefore, we selected Δ_1_ for the analyses involving Chinese hare (n = 13–53 for each season) and sambar (n = 8–53 for each season), and used Δ_4_ for the rest of the species. Confidence interval was evaluated by 10,000 bootstrapped replicates. Calculation was conducted with “CamtrapR” package^62^ in program R with a smoothing parameter of 1.25 for Δ_1_ and 1.00 for Δ_4_. We defined the seasonal difference as significant if the 95% CI of two seasonal Δ did not overlap.

## Supplementary information

Supplementary information 1
