# Supplementary material for: Spatial and temporal relationship between native mammals and free-roaming dogs in a protected area surrounded by a metropolis
Source: Sci Rep. 2019 Jun 3;9:8161. doi: 10.1038/s41598-019-44474-y (PMC6546781; doi:10.1038/s41598-019-44474-y)
Supplement: Supplementary file 1 — Supplementary information 1 [file 41598_2019_44474_MOESM1_ESM.docx]

**Spatial and temporal relationship between native mammals and free-roaming dogs in a protected area surrounded by a metropolis**

Shih-Ching Yen^1^, Yu-Ten Ju^2^, Pei-Jen Lee Shaner^2^, Hsiang Ling Chen^3*^

^1^ Center for General Education, National Tsing Hua University, No. 101, Section 2, Kuang-Fu Road, Hsinchu 300, Taiwan

^2^ Department of Animal Science and Technology, National Taiwan University, 50, Lane 155, Sec. 3, Keelung Rd., Taipei 106, Taiwan

^2^ School of Life Science, National Taiwan Normal University, 88, Sec. 4, Ting-Chow Rd, Taipei 116, Taiwan

^3^ Department of Forestry, National Chung Hsing University, 145, Xingda Rd., Taichung 402, Taiwan

* Corresponding author: Hsiang Ling Chen

E-mail: hsiangling@dragon.nchu.edu.tw

Tel: +886 4-2234-0345#153

Address: 145, Xingda Rd., Taichung 402, Taiwan

**Supplementary Information 1** Species names, conservation status, number of detections, total number of sites of detections for each species (site)

| **Common name** | **Scientific name** | **IUCN red list status** | **Taiwan red list status** | **Detection** | **Site (N= 121)** |
| --- | --- | --- | --- | --- | --- |
| Chinese ferret badger | *Melogale moschata* | Least concern | Least concern | 1,209 | 86 |
| Small Indian civet | *Viverricula indica* | Least concern | Nationally vulnerable | 221 | 60 |
| Chinese hare | *Lepus sinensis* | Least concern | Least concern | 93 | 9 |
| Formosan macaque | *Macaca cyclopis* | Least concern | Least concern | 396 | 45 |
| Chinese Reeve's muntjac | *Muntiacus reevesi* | Least concern | Least concern | 3,056 | 60 |
| Masked palm civet | *Paguma larvata* | Least concern | Least concern | 547 | 69 |
| Chinese pangolin | *Manis pentadactyla* | Critically endangered | Nationally vulnerable | 9 | 6 |
| Sambar | *Rusa unicolor* | Vulnerable | Nationally near threatened | 90 | 18 |
| Wild boar | *Sus scrofa* | Least concern | Least concern | 354 | 70 |

Reference

1 The IUCN Red List of Threatened Species. Version 2016-3. <www.iucnredlist.org>. Downloaded on 07 February 2017.

2 The Red List of Terrestrial Mammals of Taiwan, 2017. Endemic Species Research Institute and Forestry Bureau, Council of Agriculture, Executive Yuan, Nantou, Taiwan.

**Supplementary Information 2 Species-specific probabilities of occurrence and detection**

Mean, standard deviation (SD) and 95% confidence intervals (CI) for estimates of species-specific probabilities of occurrence and detection, and covariate effects on detection (β).

| Species | Occupancy | | | | Detection | | | |
| --- | --- | --- | --- | --- | --- | --- | --- | --- |
|  |  |  |  |  |  |  |  |  |
|  | *Mean* | *SD* | *95% CI* | | *Mean* | *SD* | *95% CI* | |
| Chinese ferret badger | 0.76 | 0.56 | 0.67 | 0.84 | 0.36 | 0.52 | 0.31 | 0.40 |
| Small Indian civet | 0.62 | 0.57 | 0.50 | 0.73 | 0.16 | 0.53 | 0.13 | 0.20 |
| Chinese hare | 0.06 | 0.61 | 0.02 | 0.12 | 0.30 | 0.60 | 0.16 | 0.48 |
| Formosan macaque | 0.39 | 0.56 | 0.29 | 0.50 | 0.28 | 0.54 | 0.22 | 0.34 |
| Chinese Reeve's muntjac | 0.43 | 0.56 | 0.32 | 0.54 | 0.63 | 0.54 | 0.57 | 0.70 |
| Masked palm civet | 0.69 | 0.57 | 0.57 | 0.80 | 0.16 | 0.55 | 0.11 | 0.21 |
| Chinese pangolin | 0.07 | 0.66 | 0.02 | 0.23 | 0.06 | 0.68 | 0.01 | 0.18 |
| Sambar | 0.13 | 0.59 | 0.07 | 0.22 | 0.17 | 0.59 | 0.09 | 0.29 |
| Wild boar | 0.70 | 0.57 | 0.58 | 0.82 | 0.15 | 0.54 | 0.11 | 0.20 |

**Supplementary Information 3 Species-specific covariate effects on occurrence**

Mean, standard deviation (SD) and 95% confidence intervals (CI) for estimates of species-specific probabilities covariate effects on occurrence (α).

| Species | Distance to settlement (α 1) | | | | RAI of dogs (α 2) | | | | RAI of cats (α 3) | | | | RAI of dogs^2^ (α 4) | | | | RAI of cats^2^ (α 5) | | | |
| --- | --- | --- | --- | --- | --- | --- | --- | --- | --- | --- | --- | --- | --- | --- | --- | --- | --- | --- | --- | --- |
|  |  |  |  |  |  |  |  |  |  |  |  |  |  |  |  |  |  |  |  |  |
|  | *Mean* | *SD* | *95% CI* | | *Mean* | *SD* | *95% CI* | | *Mean* | *SD* | *95% CI* | | *Mean* | *SD* | *95% CI* | | *Mean* | *SD* | *95% CI* | |
| Chinese ferret badger | 0.27 | 0.20 | -0.18 | 0.61 | 3.03 | 0.88 | 1.37 | 4.75 | 0.76 | 0.74 | -0.59 | 2.27 | -3.44 | 0.89 | -5.20 | -1.77 | -0.64 | 0.76 | -2.15 | 0.80 |
| Small Indian civet | 0.38 | 0.18 | 0.02 | 0.74 | 3.11 | 0.88 | 1.42 | 4.86 | 0.44 | 0.72 | -0.99 | 1.80 | -3.34 | 0.89 | -5.12 | -1.62 | -0.69 | 0.74 | -2.09 | 0.79 |
| Chinese hare | 0.48 | 0.20 | 0.14 | 0.95 | 3.31 | 0.90 | 1.61 | 5.12 | 0.25 | 0.79 | -1.39 | 1.73 | -3.18 | 0.89 | -4.96 | -1.47 | -1.13 | 0.99 | -3.37 | 0.51 |
| Formosan macaque | 0.36 | 0.15 | 0.03 | 0.64 | 3.29 | 0.89 | 1.64 | 5.03 | 0.69 | 0.71 | -0.70 | 2.07 | -3.24 | 0.88 | -4.94 | -1.57 | -0.47 | 0.78 | -1.99 | 1.10 |
| Chinese Reeve's muntjac | 0.44 | 0.15 | 0.15 | 0.77 | 3.04 | 0.87 | 1.39 | 4.74 | 0.24 | 0.72 | -1.17 | 1.64 | -3.42 | 0.89 | -5.14 | -1.73 | -1.18 | 0.90 | -3.06 | 0.42 |
| Masked palm civet | 0.35 | 0.17 | -0.02 | 0.67 | 3.27 | 0.88 | 1.65 | 5.02 | 0.78 | 0.74 | -0.61 | 2.35 | -3.22 | 0.90 | -5.02 | -1.52 | -0.52 | 0.79 | -2.01 | 1.05 |
| Chinese pangolin | 0.34 | 0.21 | -0.14 | 0.72 | 3.03 | 0.92 | 1.22 | 4.81 | 0.29 | 0.83 | -1.44 | 1.84 | -3.44 | 0.93 | -5.31 | -1.70 | -1.04 | 0.98 | -3.31 | 0.66 |
| Sambar | 0.61 | 0.24 | 0.26 | 1.17 | 2.96 | 0.89 | 1.24 | 4.70 | 0.47 | 0.74 | -0.94 | 1.96 | -3.46 | 0.92 | -5.32 | -1.74 | -1.00 | 0.88 | -2.90 | 0.56 |
| Wild boar | 0.41 | 0.19 | 0.03 | 0.83 | 3.13 | 0.88 | 1.43 | 4.84 | 0.53 | 0.72 | -0.87 | 1.96 | -3.31 | 0.87 | -5.02 | -1.63 | -0.71 | 0.81 | -2.26 | 0.90 |

**Supplementary Information 4 Species-specific covariate effects on detection probability**

Mean, standard deviation (SD) and 95% confidence intervals (CI) for estimates of species-specific probabilities covariate effects on detection (β).

| Species | Cuddeback (β1) | | | | Reconyx (β2) | | | | RAI of dogs (β3) | | | | RAI of cats (β4) | | | |
| --- | --- | --- | --- | --- | --- | --- | --- | --- | --- | --- | --- | --- | --- | --- | --- | --- |
|  | *Mean* | *SD* | *95% CI* | | *Mean* | *SD* | *95% CI* | | *Mean* | *SD* | *95% CI* | | *Mean* | *SD* | *95% CI* | |
| Chinese ferret badger | -0.65 | 0.26 | -1.17 | -0.17 | -0.43 | 0.11 | -0.66 | -0.23 | 0.11 | 0.10 | -0.07 | 0.31 | -0.08 | 0.09 | -0.25 | 0.09 |
| Small Indian civet | -0.61 | 0.39 | -1.48 | 0.03 | -0.50 | 0.16 | -0.83 | -0.21 | 0.12 | 0.16 | -0.20 | 0.43 | 0.28 | 0.12 | 0.04 | 0.51 |
| Chinese hare | -0.12 | 0.46 | -1.00 | 0.92 | -0.29 | 0.33 | -0.88 | 0.42 | 0.23 | 0.23 | -0.22 | 0.68 | 1.84 | 0.72 | 0.52 | 3.30 |
| Formosan macaque | -0.25 | 0.31 | -0.91 | 0.36 | -0.57 | 0.17 | -0.93 | -0.24 | -0.10 | 0.16 | -0.41 | 0.21 | -0.25 | 0.13 | -0.51 | -0.01 |
| Chinese Reeve's muntjac | -0.05 | 0.28 | -0.58 | 0.53 | -0.52 | 0.14 | -0.81 | -0.27 | 0.52 | 0.13 | 0.27 | 0.79 | 0.99 | 0.17 | 0.66 | 1.32 |
| Masked palm civet | 0.01 | 0.28 | -0.51 | 0.59 | 0.02 | 0.21 | -0.37 | 0.42 | -0.41 | 0.15 | -0.70 | -0.12 | -0.21 | 0.14 | -0.50 | 0.05 |
| Chinese pangolin | -0.34 | 0.56 | -1.55 | 0.77 | -0.39 | 0.36 | -1.16 | 0.40 | 0.39 | 0.63 | -0.52 | 1.89 | 0.39 | 1.03 | -1.66 | 2.55 |
| Sambar | -0.20 | 0.42 | -1.01 | 0.72 | -0.34 | 0.32 | -0.95 | 0.32 | -0.22 | 0.35 | -0.95 | 0.41 | 1.08 | 0.43 | 0.23 | 1.93 |
| Wild boar | -0.12 | 0.29 | -0.67 | 0.49 | -0.27 | 0.17 | -0.58 | 0.08 | 0.28 | 0.12 | 0.05 | 0.51 | -0.34 | 0.16 | -0.65 | -0.03 |

**Supplementary Information 5 Diel activity pattern of the native mammals and invasive carnivores.**

The diel activity was estimated by kernel density estimation on circular data. A higher density represents increased activity.


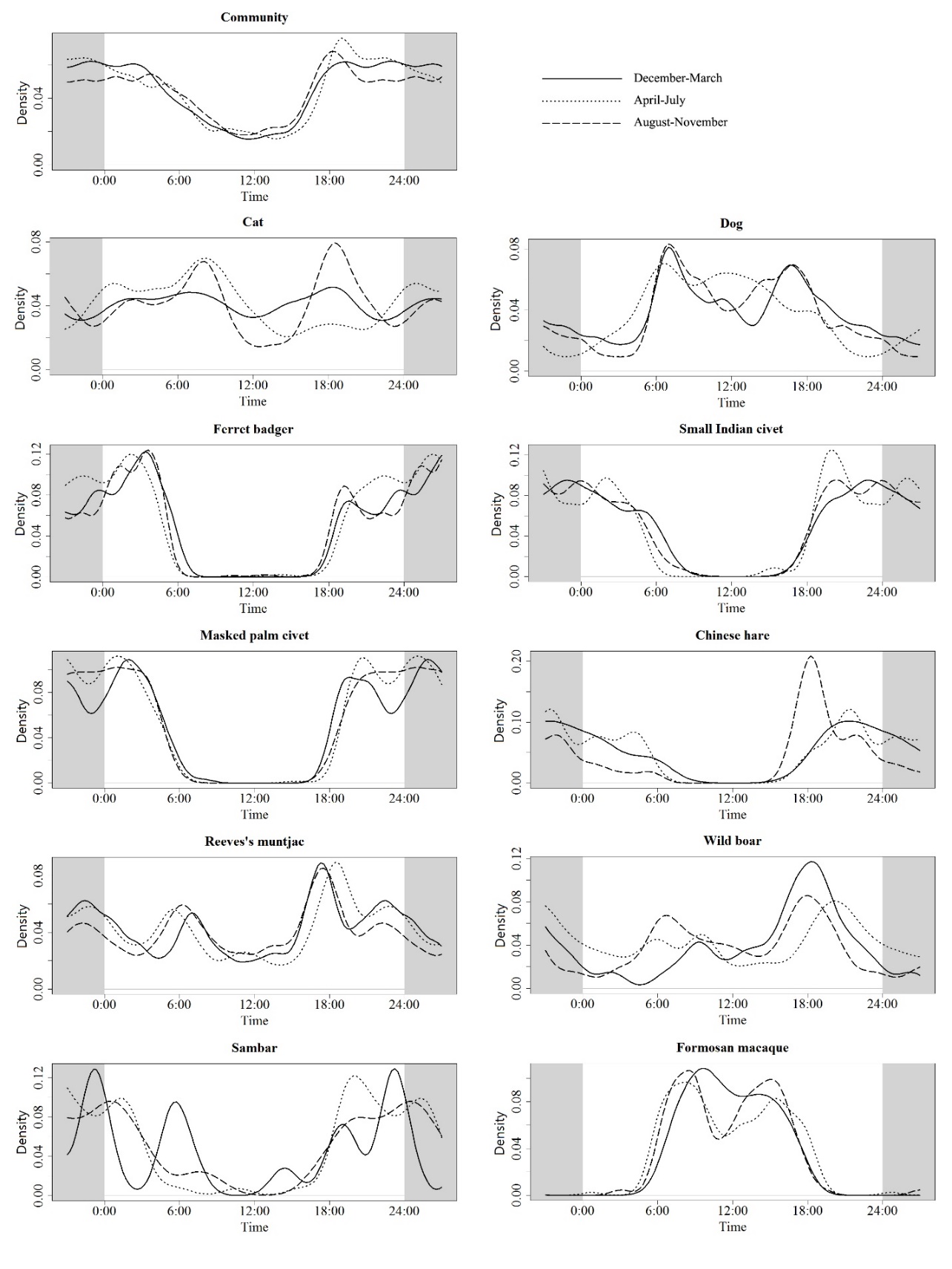


**Supplementary Information 6**

To avoid having too many non-detection records^1,2^, we defined a sampling period as an interval of seven days (a repeat survey), in which the species was detected or not detected at a camera site, resulting in an average of 20 surveys (SE = 1.6) per camera station from March 2012 to July 2017. We estimated the probability of observing species *i* at camera station *j* during survey *k* conditional on the site being occupied as x *_i,j,k_*~ Bern(p *_i,j,k_* * z *_i,j_*). The detection probability, p*_i,j,k_* , was the probability species *i* was detected at camera station *j* during survey *k*. The occurrence, z*_i,j_*, was a latent binary variable where z*_i,j_* = 1 if camera station *j* was used by species *i* and zero if not. The occurrence was modeled as a Bernoulli random variable, z*_i,j_* ~Bern(ψ*_ij_*), where ψ*_ij_* is the probability that species *i* occurred at camera station *j*^3^. We modeled occupancy and detection to be jointly distributed^4^. We linked species-speciﬁc occupancy models to a community-level occupancy model by treating individual species as random effects derived from a normally distributed, community-level hyper-parameter^5,6^. Hyper-parameters specify the mean response and variation among species within the community to a covariate^7^. For the community model, the α coefficients were modelled as α~ normal (µ_α_, σ^2^_α_) where µ_α_ is the community-level mean and σ^2^_α_ is the variance^8^. For priors, we used a uniform distribution of zero to one on the real scale for α0 and β0 and uniform from zero to 10 for σ parameters. We used a normal prior distribution with a mean of zero and standard deviation of 100 on the logit-scale for the remaining covariates^5^.

References:

1 Mackenzie, D. I. & Royle, J. A. Designing occupancy studies: general advice and allocating survey effort. *J. Appl. Ecol.* **42**, 1105-1114; 10.1111/j.1365-2664.2005.01098.x (2005).

2 Tobler, M. W., Zúñiga Hartley, A., Carrillo-Percastegui, S. E. & Powell, G. V. N. Spatiotemporal hierarchical modelling of species richness and occupancy using camera trap data. *J. Appl. Ecol.* **52**, 413-421; 10.1111/1365-2664.12399 (2015).

3 MacKenzie, D. I. *et al.* Estimating site occupancy rates when detection probabilities are less than one. *Ecology* **83**, 2248-2255; 10.1890/0012-9658(2002)083[2248:ESORWD]2.0.CO;2 (2002).

4 Dorazio, R. M. & Royle, J. A. Estimating size and composition of biological communities by modeling the occurrence of species. *Journal of the American Statistical Association* **100**, 389-398 (2005).

5 Rich, L. N. *et al.* Using camera trapping and hierarchical occupancy modelling to evaluate the spatial ecology of an African mammal community. *J. Appl. Ecol.* **53**, 1225-1235; 10.1111/1365-2664.12650 (2016).

6 Zipkin, E. F., Andrew Royle, J., Dawson, D. K. & Bates, S. Multi-species occurrence models to evaluate the effects of conservation and management actions. *Biol. Conserv.* **143**, 479-484; doi.org/10.1016/j.biocon.2009.11.016 (2010).

7 Kéry, M. & Royle, J. A. Hierarchical Bayes estimation of species richness and occupancy in spatially replicated surveys. *J. Appl. Ecol.* **45**, 589-598; 10.1111/j.1365-2664.2007.01441.x (2008).

8 Chandler, R. B. & Royle, J. A. Spatially explicit models for inference about density in unmarked or partially marked populations. *The Annals of Applied Statistics* **7**, 936-954; 10.1214/12-aoas610 (2013).

**Supplementary Information 7**

We performed preliminary LOESS regressions to explore the most likely forms of relationships between species richness of native mammals and distance to human settlements, dog RAI (relative activity level index) or cat RAI for constructing statistical models. The grey lines denote no smoothing, and the red, orange, purple, green and blue lines denote different levels of smoothing (from red to blue, span = 0.1, 0.25, 0.5, 0.75, 0.9). The left panel shows LOESS regressions on full data sets, and the right panel on the subsets of no zeros.

**
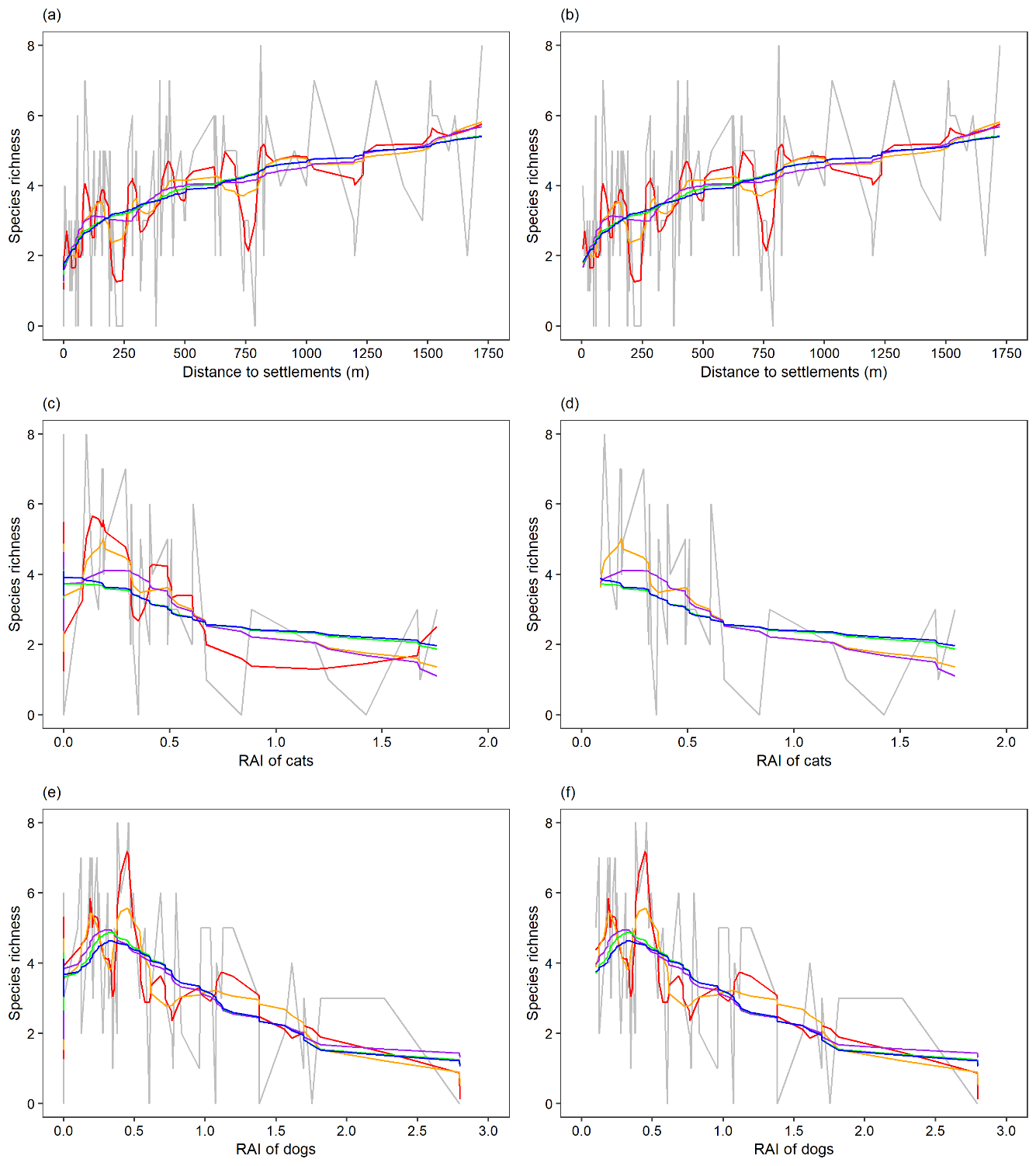
**

**Supplementary Information 8**

The model code below is based on the code written by Rich et al. (2016) for program R and uses the R2jags package to run JAGS. The data file, ‘Data_civet’, includes columns representing the study site, species (SppCode), number of surveys during which the species was photographed ≥1 time (detections), number of sampling occasions (num.survey) and covariate values.

#Define the covariates for occupancy X = Data_civet[,c(10,13:16)]

#Define the covariates for detection dX = Data_civet[,c(8:9,13:14)])

#Load the necessary libraries

library(R2jags); library(reshape); library(plyr)

#Define the necessary arguments to run the jags command

#Load all the data including the detection array, number of sampling occasions, individual #species sampled, total number of sampled species, and covariate information

data <- list(D = Data_civet$detections, N = ceiling(Data_civet[,"num.survey"]), Species=

as.numeric(Data_civet$SppCode), n = nrow(Data_civet),

nspp = max (as.numeric(Data_civet$SppCode)),X = X, dX = dX)

#Specify the initial values

inits = function() {list(Z = as.numeric(data$D>0))}

#Specify the parameters to be monitored

params = c("rho","pbeta","spbeta","sigpbeta","mbeta","sigbeta","sbeta",

"psi.mean","sigma.occ","p.mean","sigma.p","alpha","Z","P")

#Specify the number of chains (nc), number of iterations (ni), burn-in period (nb), and thinning

#rate (nthin)

nc = 3

ni = 60000

nb = 10000

nthin = 50

cat("

data {

nBeta <- dim(X)

nP <- dim(dX)

}

model {

# Define covariance parameter between detection and mean occupancy

rho ~ dunif(-1,1)

var.p <- sigma.p /(1.-pow(rho,2))

#Define prior distributions for occupancy parameters

alpha.mean <- log(psi.mean) - log(1-psi.mean)

psi.mean ~ dunif(0,1)

sigma.occ ~ dunif(0,10)

tau.occ <- pow(sigma.occ,-2)

#Define prior distributions for true positive detections

p.mean ~ dunif(0,1)

b <- log (p.mean) - log(1-p.mean)

sigma.p ~ dunif(0,10)

tau.p <- pow (sigma.p,-2)

#Define prior distributions for occupancy effects where nbeta is the number of occupancy

#covariates in the model, mbeta is the community-level hyper-parameter for each of the nbeta

#covariates, tbeta is the amount of variability in each of the community-level hyper-

#parameters, and sbeta is the species-specific covariate effects

for (a in 1:nBeta[2]){

mbeta[a] ~ dnorm(0,0.01)

sigbeta[a] ~ dunif(0,10)

tbeta[a] <- pow(sigbeta[a],-2)

for (i in 1:(nspp)) {

sbeta[i,a] ~ dnorm(0,tbeta[a])

}

}

#Define prior distributions for detection effects where nP is the number of detection covariates in

#the model, pbeta is the community-level hyper-parameter for each of the nP covariates, tpbeta is

#the amount of variability in each of the community-level hyper-parameters, and spbeta is the

#species-specific covariate effects

for (a in 1:nP[2]){

pbeta[a] ~ dnorm(0,0.01)

sigpbeta[a] ~ dunif(0,10)

tpbeta[a] <- pow(sigpbeta[a],-2)

for (i in 1:(nspp)) {

spbeta[i,a] ~ dnorm(0,tpbeta[a])

}

}

#Define prior distributions for the occupancy and detection covariates for each species

for (i in 1:(nspp)) {

alpha[i] ~ dnorm(alpha.mean, tau.occ)

mu.p[i] <- b + (rho*sigma.p /sigma.occ)*(alpha[i] - alpha.mean)

P[i] ~ dnorm(mu.p[i], var.p)

}

#Estimate the occupancy probability (latent Z matrix) for each species at each camera station

for (j in 1:n) {

logit(psi[j]) <- alpha[Species[j]] + inprod(mbeta,X[j,])+ inprod(sbeta[Species[j],],

X[j,])

#Estimate the detection probability for each species at each camera station

logit(p[j]) <- P[Species[j]] + inprod(pbeta,dX[j,]) + inprod(spbeta[Species[j],],

dX[j,])

Z[j] ~ dbern(psi[j])

zp[j] <- p[j]*Z[j]

D[j] ~ dbin(zp[j], N[j])

}

}

#Finish writing the text file

", file = "AllMammals.txt")

#Run the model and call the results "output"

output<- jags(data = data, inits = inits, parameters.to.save = params, model.file

="AllMammals.txt", n.chains =nc, n.iter =ni, n.burnin =nb, n.thin =nthin)

#See a summary of the parameter estimates

output.sum <- output$BUGSoutput$summary

write.csv(output.sum,file="output.csv")

#-------------------------------------------------Occupancy

#Define the occupancy covariate effects where mbeta is the community-level hyper-parameter,

#and sbeta is the species-specific parameter

mbeta <- output$BUGSoutput$sims.list$mbeta

sbeta <- output$BUGSoutput$sims.list$sbeta

#TO ESTIMATE SPECIES-SPECIFIC COVARIATE VALUES:

#Begin by defining the species

spec <- Spp[,1]

#Define the covariates

covs <- colnames(X)

#Create a data frame where the number of rows is equal to the number of covariates * the

#number of species

species <- data.frame(expand.grid(covs,spec), matrix(NA,length(covs)*length(spec),4))

colnames(species) <- c("Covariate","Species","Mean","SD","LCI","UCI")

#Create a loop that will estimate species-specific values for each of the covariates

for (a in 1:length(covs)){

for (b in 1:length(spec)){

sims <- mbeta[,a] + sbeta[,b,a]

species[(5*(b-1)+a),3:6] <- c(mean(sims), sd(sims),

quantile(sims,c(0.025,0.975)))

}

}

#Export the results as a table

write.csv(species,file="Results_species.csv")

#------------------------------------------------Detection

#Define the detection covariate effects where mbeta is the community-level hyper-parameter,

#and spbeta is the species-specific parameter

pbeta <- output$BUGSoutput$sims.list$pbeta

spbeta <- output$BUGSoutput$sims.list$spbeta

#TO ESTIMATE SPECIES-SPECIFIC COVARIATE VALUES:

#Define the covariates

covs <- colnames(dX)

#Create a data frame where the number of rows is equal to the number of covariates * the

#number of species

species <- data.frame(expand.grid(covs,spec), matrix(NA,length(covs)*length(spec),4))

colnames(species) <- c("Covariate","Species","Mean","SD","LCI","UCI")

#Create a loop that will estimate species-specific values for each of the covariates

for (a in 1:length(covs)){

for (b in 1:length(spec)){

sims <- pbeta[,a] + spbeta[,b,a]

species[(4*(b-1)+a),3:6] <- c(mean(sims), sd(sims),

quantile(sims,c(0.025,0.975)))

}

}

#Export the results as a table

write.csv(species,file="Results_species_detection.csv")

#TO ESTIMATE SPECIES RICHNESS FOR EACH SITE:

#Define the z matrix

z = output$BUGSoutput$sims.list$Z

#Sort the data frame based on species, study site

d <- Data_civet[,c(1:2)]

#Create a new data frame

dz <- data.frame(d,t(z))

#Melt the data frame for easy casting

m.dz <- melt(dz,id.vars = c("Plot_ID","SppCode"))

#Aggregate the data by summing the values in the z matrix for each camera station during each

#iteration

z.all <- acast(m.dz, Plot_ID ~ variable, fun.aggregate = sum)

#Use the aggregated values to create probability distributions and estimate mean, sd, and 95%

#credible interval values for camera-station specific species richness

z.all <- t(apply(z.all,1,function(x) c(mean(x),sd(x),quantile(x,c(0.025,0.975)))));

colnames(z.all) = c("Mean","SD","LCI","UCI")

#Export estimates of species richness as a table

write.csv(z.all,file="Results_spprich.csv")

Reference:

1. Rich, L. N. et al. Using camera trapping and hierarchical occupancy modelling to evaluate the spatial ecology of an African mammal community. J. Appl. Ecol. 53, 1225-1235; 10.1111/1365-2664.12650 (2016).
